# Data Analysis in Newly Developed Milk Sensor Platforms: Good Practices, Common Pitfalls, and Hard-Earned Lessons from Field Application

**DOI:** 10.3390/foods14101724

**Published:** 2025-05-13

**Authors:** Francesco Martelli, Claudia Giacomozzi, Roberto Dragone, Chiara Frazzoli, Gerardo Grasso

**Affiliations:** 1 Dipartimento Malattie Cardiovascolari ed Endocrino-Metaboliche, e Invecchiamento, Istituto Superiore di Sanità, Via Giano Della Bella, 34, 00162 Rome, Italy; francesco.martelli@iss.it (F.M.); claudia.giacomozzi@iss.it (C.G.); chiara.frazzoli@iss.it (C.F.); 2 Istituto per Lo Studio Dei Materiali Nanostrutturati Sede Sapienza, Consiglio Nazionale delle Ricerche, P. le Aldo Moro 5, 00185 Rome, Italy; roberto.dragone@cnr.it

**Keywords:** animal welfare, food safety, milk chain, milk monitoring, sensors

## Abstract

In the last decade, the demand for healthier and safer food has increased alongside greater consumer awareness of food consumption, particularly in developed countries. This trend has pushed the food industry to implement a wide range of food quality control measures and surveillance systems for detecting contaminants. While high-end laboratory techniques remain the gold standard detection techniques, there is a growing need for simpler, more robust diagnostic tools that can be applied in the early stages of the food production chain to promptly identify deviations that may compromise food safety or quality. A complementary approach using both techniques can result in an enhancement of the overall contaminant-detection effectiveness and a better balance between food safety decision-making and the preservation of production value. This need is particularly relevant in farming and in the dairy industry. Developing milk process analytics requires careful consideration of both the nature of the processed sample and the conditions under which it is collected. Moreover, newly introduced techniques require the development of sound methodologies for data collection, analysis, and statistical process control. For this reason, this paper presents a detailed analysis of our previous milk data-collection campaigns involving technological prototypes, aiming to identify and suggest ways to preventively minimize issues related to experimental data collection, interpretation, errors, and mishandling. This analysis resulted in a set of practical observations and recommendations reported in the paper.

## 1. Introduction

In the last decade, several drivers have pushed the food industry to implement a wide range of food quality and safety controls. Demand for healthier and safer food has risen in parallel with the increase in food-consumption consciousness, mainly in developed countries but also in emerging ones [1,2,3,4,5]. In the meantime, other factors such as climate change [6], emerging contaminants in food [1,2,6,7], or water used for crop irrigation [8] or for animal farming have come into play, pushing for and justifying research work aimed at the development of a new class of measurement and detection devices, in order to deliver fast and accurate detection of sample’s characteristics and/or contamination sources, in a cost-effective way [9]. While high-end techniques such as mass spectrometry (MS) and tandem mass spectrometry (MS/MS), enzyme-linked immunosorbent assay (ELISA) and nanomaterials-based ELISA, gas chromatography (GC), high performance liquid chromatography (HPLC) and quantitative real-time polymerase chain reaction (qPCR) are and will remain the gold standard detection techniques [10,11,12,13,14,15,16], simpler and robust diagnostic devices are needed from the earliest phases of the food production chain, e.g., capable of routine screening of food samples for the detection of targeted contaminants [17,18,19]. In the dairy supply chain, milk analyses are not routinely performed at the farm level; instead, they are typically conducted offline in laboratories. The availability of cost-effective, sensor-based devices for routine, on-site monitoring of food productions—including milk—could significantly contribute to minimizing food waste, reducing inequalities through sustainable development, and identifying sample outliers for confirmatory analyses. Moreover, such tools could facilitate the early detection of process deviations that may lead to unsafe or lower-quality food products [20,21,22]. As an illustrative example, the illegal adulteration of milk products with melamine is typically analyzed using mass spectrometry techniques. However, recent studies highlight the potential of nanoparticle-based colorimetric sensors, particularly those utilizing silver and gold nanoparticles, for the low-cost, on-site detection of melamine in milk. These sensors enable rapid, visible identification of melamine adulteration, and their integration into routine monitoring systems could be beneficial for improving food safety and reducing waste [23,24,25,26,27].

Furthermore, sensor-based technologies can complement and enhance laboratory analytical techniques, such as MS, which is effective for analyzing milk components like proteins, lipids, and metabolites as well as for the detection of contaminants like mycotoxins, veterinary drug residues, pesticides, and persistent organic compounds [28]. The free ion content and physicochemical properties of the milk matrix, measurable by sensors, can influence the behavior of proteins and lipids, thereby affecting MS analysis results. Integrating sensor-based technologies to monitor these milk components in both field and laboratory settings can significantly complement gold standard techniques in various ways, enhancing the overall effectiveness of contaminant detection and analysis. From the workflow optimization point of view, sensor-based devices can serve as rapid, preliminary screening tools that identify potential contaminants before samples are sent to laboratories for MS analysis. For instance, a sensor might detect elevated levels of chemical contaminants in milk, allowing for immediate action to be taken, such as recalling a batch or conducting further testing. This initial screening can significantly reduce the number of samples that require time-consuming analysis by gold standard techniques like MS. Additionally, sensor devices can be seamlessly integrated into a real-time framework for monitoring and managing milk quality parameters throughout production and processing. This allows continuous data collection and, consequently, nearly immediate adjustments to production processes, ensuring that any deviations from safety standards are addressed promptly. By integrating sensor data with laboratory results, producers can establish a more robust quality and safety control system, enhancing food safety and minimizing the risk of contamination reaching consumers.

From a data interpretation perspective, this complementary approach is particularly valuable. Integrating the large volume of sensor data with MS results enables continuous monitoring of potential contaminants, functioning as an early warning system, while MS provides detailed molecular characterization. This synergy supports a more comprehensive understanding of contamination profiles, improving the balance between informed food safety decision-making and the preservation of product value.

There is growing interest in microfluidic devices [29,30,31,32,33,34] whose technological features show considerable promise, particularly for applications involving fluid samples. This is especially relevant in farming and in the dairy industry, which represent a significant share of agricultural production and play a crucial role in human nutrition in many countries [35]; at the same time, milk and dairy production lines can benefit from early-warning signals [36], enabling timely interventions to maintain product quality and safety.

To address this need, the authors were recently involved in two different development projects focused on milk production monitoring. The first, the ALERT Project, involved an early-warning multi-sensor system designed to monitor biological, chemical, and physicochemical parameters. The second, the MOLOKO Project, focused on the development of an automatic, miniaturized, integrated photonic sensor.

Both projects aimed to develop fully engineered sensors (or sensor platforms) capable of detecting a range of raw milk parameters and contaminants, while maintaining certain operational characteristics. These characteristics include ease of execution, no chemical pretreatment of raw cow’s milk samples, adequate analytical performance in harsh environments, minimal operator training requirements, and rapid detection of anomalies in milk.

Findings from the ALERT Project quickly demonstrated that the introduction of new techniques must be accompanied by the development of sound methodologies for data collection, analysis, and statistical process control [37]. This aspect is of great relevance in the context of milk and dairy products, which are complex food matrices naturally rich in nutrients and bioactive compounds.

Analyzing cow’s milk composition is a challenging task, especially when conducted directly on the dairy farms. As a complex fluid composed of carbohydrates, simple and complex lipids, proteins, and minerals in aqueous phase, raw milk analysis typically requires sample preparation and/or extraction to minimize potential interference among its constituents [38]. In addition to the natural variability of the sample itself, various factors—such as sampling methods, sample conservation, and preparation—can also affect measurements. To address potential uncertainties arising from sample conservation and preparation [39], in-line analytics have become a central focus of recent research [40]. However, the development of milk process analytics—whether in-line, online or at-line—requires particular attention due to the complex nature of the sample and the environment from which the sample is taken.

In addition to challenges associated with laboratory measurement techniques, it is important to note that the development of new technologies often requires cooperation among diverse and multidisciplinary teams. This add an additional layer of complexity, as the frequent exchange of milk samples, hardware prototypes, and data analysis methods necessitates additional and coordinated effort to ensure that teams conduct comparable tests on equivalent samples, use identical hardware setups consistently, and follow standardized data analysis procedures.

For this reason, in the MOLOKO project a detailed analysis of milk data collection campaigns involving technological prototypes has been carried out, with the aim of identifying and proactively minimizing issues, errors, and mishandling during experimental data collection.

This analysis led to a set of practical observations and recommendations aimed at accelerating data collection and analysis, enhancing cost-effectiveness, and improving data reliability and consistency.

## 2. Materials and Methods

The experiences described in this work derive from the two projects, here briefly described.

### 2.1. The ALERT Project

**Figure 1:**
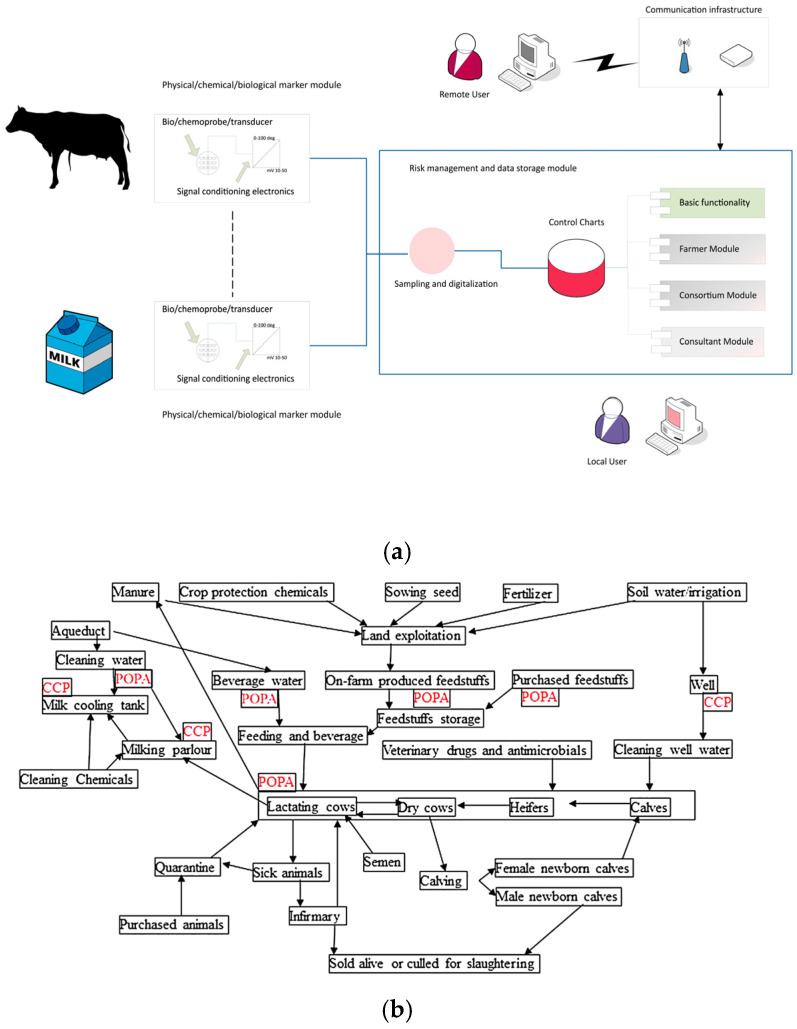
(**a**) Schematic representation of the operational functioning of the BEST platform. (**b**) General flow diagram of CCPs and POPAs in the dairy farm production process [42].

The ALERT project, titled “Integrated system of bio-sensor and sensor (BEST) for monitoring of wholesomeness and quality, as well as for traceability in the cow milk chain”, was funded by the Italian Ministry of Economic Development (Industria 2015 New technologies for Made in Italy, grant MI 00195). The project developed and employed the semi-automated BEST platform (European Patent EP2304428B1) [41], a HACCP-like multi-sensor early warning system for the simultaneous and continuous monitoring of sets of biological, chemical, and physio-chemical parameters in food matrixes (Figure 1a). The BEST platform is suitable for toxicological self-monitoring, diagnostics, and traceability in food-producing systems, enabling the early identification, control, monitoring, and management of anomalous variations in selected (bio)markers—either at Critical Control Points (CCPs) or other Points of Particular Attention (POPAs) (Figure 1b). 

In an HACCP-based integrated chain approach, Critical Control Points (CCPs) are mandatory monitoring stages, as failures at these points can lead to serious consequences, including production interruptions and product loss (e.g., milk waste). In contrast, according to the design of the BEST platform, Points of Particular Attention (POPAs) are non-mandatory locations within the process where measurable anomalous trends may occur. While not required, POPAs provide producers with a more nuanced understanding and management of potential anomalies or deviations from expected values. These deviations, detected through variations in relevant control charts, can trigger early risk management procedures within HACCP-like frameworks. This approach enables earlier intervention, thereby enhancing overall process control and reducing the likelihood of reaching critical failure points. More specifically, the BEST platform was utilized in the ALERT project for sampling and sensor-based, in-field analyses of individual cow’s raw milk on a dairy farm in the Lazio region of Central Italy (41°54′47.94″ N, 12°15′48.25″ E). The selected dairy farm for the present study adhered to good standards of dairy farming, agricultural practices, and sanitary protocols [42]. The compositional and physio-chemical parameters measured were temperature (°C); dissolved gasses such as oxygen (O_2_, ppm) and carbon dioxyde (CO_2_, mV); redox potential (mV); pH (pH units); ionic conductivity (mS/cm); and free inorganic ion fraction, namely calcium ion (Ca^2+^, mV), ammonium ion (NH_4_^+^, mV), nitrate ion (NO_3_^−^, mV), and chloride ion (Cl^−^, mV). Free inorganic ion fraction (Ca^2+^, NH_4_^+^, NO_3_^−^, Cl^−^), CO_2_, and redox potential are here expressed as electrical potential (mV) as measured by combination ion selective electrodes (ISEs) for Ca^2+^, NH_4_^+^, NO_3_^−^, and Cl^−^ (Sentek Ltd., Braintree CM7 2SE, UK), while pH, redox potential, and ionic conductivity sensors were purchased by Amel Srl (Milan, Italy). Dissolved oxygen sensor and dissolved carbon dioxide sensors were purchased by Mettler Toledo (Milan, Italy).

These parameters have been chosen due to their well-documented interconnections and their ability to reflect variations in milk quality and safety, including changes in nutritional composition, as well as potential indicators of contamination or spoilage. They can also indicate milk adulteration, be associated with (patho)physiological conditions in dairy cows, and influence the technological properties of milk [43,44,45,46,47,48,49,50,51,52,53].

Table 1 reports the analytical characteristics of sensors integrated into the BEST platform.

Using the BEST platform prototype installed at the dairy farm in Central Italy, up to 15 individual raw milk samples were collected daily by an in-line automatic sampler installed at one of the farm’s 14 milking units in the milking parlor. A representative milk sample was obtained from each cow connected through the milking machine of that unit. This sampler, approved by the International Committee for Animal Recording [54], was installed at one of the 14 milking stations in the milking parlor. Milk samples were collected and transported to the BEST platform using a specially designed automated system. Each raw milk sample was linked to the respective cow through automatic pedometers (provided by Nutriservice Srl, Brescia, Italy) and a registration number. During the sampling period (March to June), 72 different dairy cows were randomly sampled at least once. Preliminary laboratory tests indicated that daily calibration was unnecessary, as the sensor’s daily drift was ≤1% which did not affect the daily results. These tests were conducted to verify the accuracy of each sensor using three calibration standard solutions with known analyte concentrations. The signals were plotted against the analyte concentration or, for combination ion-selective electrodes, the logarithm of the ion concentration to determine the correct slope of the calibration curves. A two-point calibration of the dissolved O_2_ sensor was performed at 25 °C by measuring O_2_ in open air, where the O_2_ concentration at 24 °C and an atmospheric pressure of 760 mmHg is 8.20 mg L^−1^, and dissolved oxygen in a 10 g L^−1^ sodium sulfite solution, where the O_2_ concentration is 0 mg L^−1^ (sodium sulfite powder ≥98%, purchased from Merck, Milan, Italy).

For anomaly detection using BEST data, mean values for each milk parameter were calculated from daily samples collected consecutively from 10 cows. These mean values exhibited a relative standard deviation (%RSD) of less than 5%, indicating high measurement consistency. At the end of each measurement cycle, the BEST platform generated a set of multiparameter data, which were analyzed using multivariate analysis using Milkcheck Beta 0.2 software, developed in a Python 3.5 virtual environment on the Ubuntu operating system (Amel Srl, Milan, Italy). During the project, the proof-of-concept of the primary objective was successfully demonstrated: the development and deployment of the BEST system environment for continuous monitoring in a real dairy farm setting. This was further supported by targeted training sessions for dairy farm operators on the routine application of the BEST technology.

### 2.2. The MOLOKO Project

**Figure 2:**
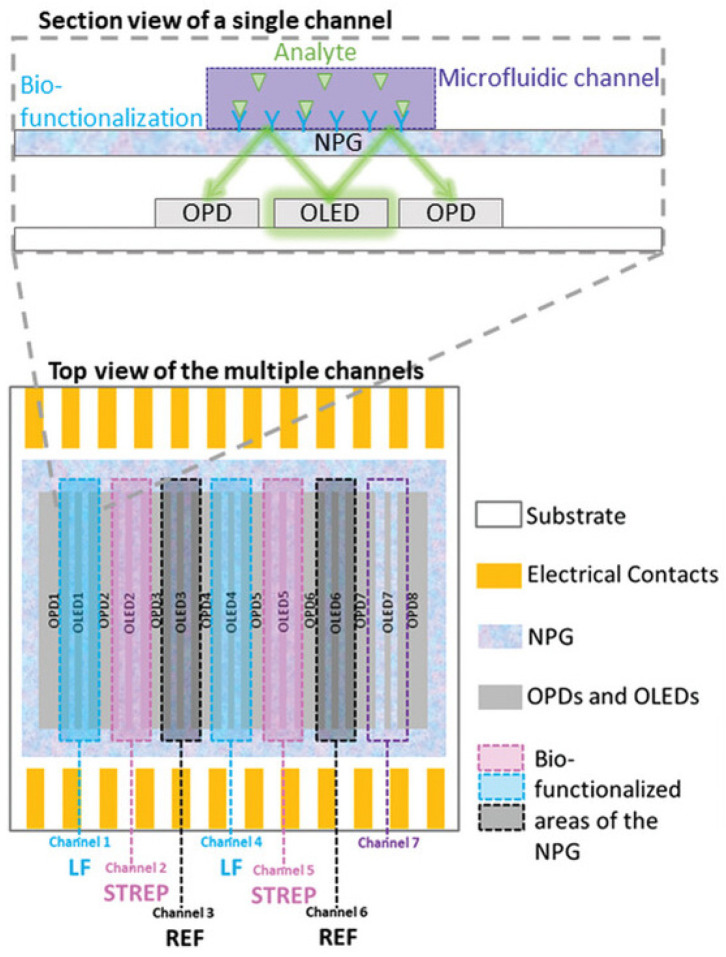
Schematic representation of the MOLOKO sensor chip (modified from [55]).

The MOLOKO project has received funding from the European Union’s Horizon 2020 research and innovation program under grant agreement no. 780839. Its main objective was the development and implementation an automatic miniaturized integrated photonic sensor (Figure 2) to be used as process analytical instrumentation for rapid, on-site monitoring of analytes relevant to safety and quality within the milk supply chain. Specifically, the project aimed at the multiplexed quantitative detection of up to 10 analytes among which were food safety parameters (e.g., antibiotics and toxins) as well as food quality parameters.

The MOLOKO project successfully achieved full integration of optoelectronic components, a microfluidic system for automated sample handling, and a user-friendly interface equipped with accompanying software and data management tools.

More specifically, the MOLOKO project results included the quantitative detection of lactoferrin and streptomycin using plasmonic sensing, achieved through the integration of organic optoelectronic devices and a nanostructured plasmonic grating. The sensor provided a quantitative and linear response. It achieved a limit of detection of 10^−^⁴ refractive index units when calibrated with standard solutions. Using a custom algorithm based on principal-component analysis, the biosensor demonstrated a limit of detection as low as 3.7 µg mL^−^^1^ for lactoferrin. For streptomycin, the sensor achieved detection of concentrations at the maximum residue level of 200 ng mL^−^^1^ set by law [55]. Another result was the development of a multiplex immunoassay for distinguishing between β-Casein A2 and β-Casein A1. The antibodies used in the assay showed high specificity for their corresponding βCA proteins, with cross-interaction between the βCA1 and βCA2 assays being less than 10% [56]. Lastly, the project activities have led to the identification of recombinant antibodies targeting different epitopes of *Staphylococcus aureus* Enterotoxin A (SEA), which are currently under patent application.

Table 2 illustrates the significant complementarities between the ALERT and MOLOKO projects, while also highlighting their unique features and distinct capabilities in independently addressing specific challenges. The ALERT project focused on the development and field deployment of the BEST technology in real dairy farm environments, enabling continuous monitoring within a broader HACCP-like, multi-sensor framework. In contrast, the MOLOKO project concentrated on the development of a miniaturized optical biosensor for the targeted detection of specific analytes, integrating plasmonic sensing with organic optoelectronic components.

## 3. Results

Both projects—ALERT and MOLOKO—included a validation phase to assess the validity and reproducibility of their respective measurements. In both projects, this phase required more resources than initially planned. This was largely due to an underestimation of the complexities of the dairy farm environment, the inherent variability in raw milk characteristics, and the unexpectedly high variability in sampling procedures and conditions. Validation activities were considerably delayed by a range of factors, including numerous minor technical issues (e.g., clogging, failures in tubing and gaskets, unexpected behavior of sensors and general instrumentation, and unstable data communication in a rural area) as well as unforeseen milk variability resulting from fluctuations in animal behavior on a dairy farm. However, the majority of the unplanned resource consumption—primarily in terms of time and personnel—stemmed from three main factors that were somehow underestimated during the projects planning phases. These are described in detail in the following sections.

### 3.1. The Need for a Settled Knowledge of Sensors and Instrumentation Behavior

For any analyzed parameter, it is crucial to have detailed practical knowledge of the sensor or instrument used, particularly regarding its technical behavior when operating with milk. This requirement also extends to ancillary equipment and fixtures, such as valves and pumps, that are designed to work with milk. However, limited practical experience may exist in design labs concerning the specific product or model, in the context of milk as a unique matrix. The primary reason for this is that the milk matrix, rich in fat and proteins in phase equilibrium, can interfere with most sensor surfaces in various ways [22]. Moreover, the structure of milk phases can be altered by the measurement process itself.

This recommendation is particularly relevant when data collection is conducted in the field, such as in a cowshed or dairy plant. A considerable allocation of resources is required for such data collection, making it essential to have an operation plan in place to ensure the process is orderly, efficient, and effective data acquisition. Proper planning helps mitigate the risk of having to repeat data collection due to discrepancies between the actual behavior of sensors or instruments and the initial expectations.

**Figure 3:**
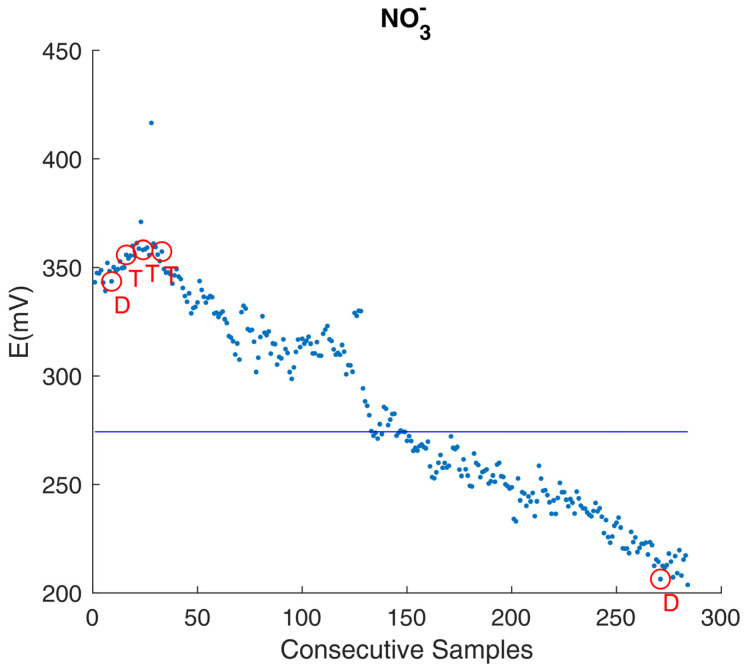
Exemplificative NO_3_^−^ combination ISE decline in performance, over a 3-month sampling time frame, despite proper maintenance. The plot contains 377 consecutive samples acquired from 118 animals in total across the three months. The NO_3_^−^ combination ISE uses a solid-state PVC membrane and a gel-filled reference electrode. Circled data are from animals with suspected health issues (T) or different physiological conditions (D). Sensor’s output is reported in mV along the vertical axis of the plot.

In the example plotted in Figure 3, an exemplificative NO_3_^−^ combination ISE exhibits a significant decline in performance over a three-month period, despite following the manufacturer’s maintenance guidelines. This suggests the potential for poisoning effects.

The unexpected sensor behavior had a direct consequence: data from animals suspected of health issues (T) or differing physiological conditions (D) were analytically indistinguishable from data from healthy animals (Figure 1, circled data points). While some degree of sensor drift was anticipated, the actual extent (>40% over 90 days) of the drift rendered it impossible to identify abnormal fluctuations in NO_3_^−^ ion concentrations that would typically signal underlying health issues.

The detrending procedure was performed as follows: for each day, the daily median sensor value was subtracted from the raw sensor readings, and the overall median sensor value, calculated across the entire three-month period, was then added.

Therefore, given

S_i_(1..n_i_) n_i_ individual milk daily sensor’s values for day i
(i = 1.. 90, n_i_ variable from 5 to 12, mean 9.09 sd = 2.02)
$m_{i}=median\left[S_{i}\left(1..n_{i}\right)\right]$         Daily median sensor value over day i
$M=median\underset{i}{⋃}\left[S_{i}\left(1..n_{i}\right)\right]$        Median sensor value over all days i
Daily detrended sensor values were calculated as:
  D_i_(1..n_i_) = S_i_(1..n_i_) − *m_i_* + *M* (1)
  The Formula (1) can be read as follows: Daily detrended sensor values = Daily sensor values − Daily median sensor value + Overall median sensor value (three-month period).

**Figure 4:**
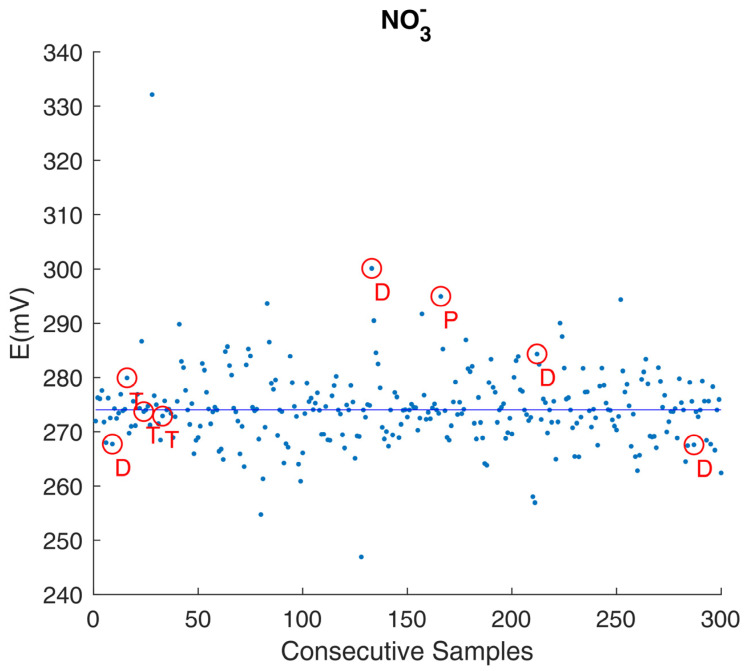
NO_3_^−^ concentration, detrended data (original data plotted in Figure 1). The detrend procedure allowed for better highlighting of animals with suspected health issues (T) or different physiological conditions (D, P), the latter clearly standing out of the average milk nitrate content.

It was only after a detailed subsequent study of sensor behavior that the feasibility of a linear detrend procedure could be demonstrated, as shown in Figure 4.

The results of the detrending procedure revealed distinct NO_3_^−^ concentration profiles in certain cases. For example, milk samples from animals of different breeds (Figure 4, D) or different post parturient stages (Figure 4, P) displayed unique NO_3_^−^ concentrations, which were not evident in Figure 3.

However, it is important to note that this detrending procedure may not be universally applicable. For instance, it would be impractical for datasets collected over longer periods (i.e., more than 2–3 months), where factors such as inherent milk seasonality [57] or lunar calendar influences [58] could introduce additional effects.

Therefore, before beginning any data collection with a sensor or measuring instrument, a comprehensive characterization study should be conducted. This study should assess the sensor’s measurement repeatability, reliability, and temporal stability with particular emphasis on the milk matrix.

In cases where no relevant studies are available for the off-the-shelf sensor being used, or if the sensor is newly developed, a detailed characterization study should be conducted before or in parallel with any field data collection efforts. This study should focus on evaluating the sensor’s reliability, temporal stability, and the impact of conditioning materials (such as rinsing solutions, preservation methods, transport, and storage media). Additionally, it should establish protocols to ensure the sensor’s reliability and stability within predefined ranges.

In the field experience reported here, an additional unexpected sensor behavior was observed, affecting sensor stability during warm-up and the conditioning of the sensor surface with the milk matrix. The sensors used were primarily combination ISEs with solid-state polymeric membranes, with the exception of the Cl^−^ ISE, which employed a solid-state crystal membrane. Dissolved oxygen was measured with a luminescent gas-sensitive probe, and carbon dioxide with a gas-permeable membrane electrode. Redox potential and ionic conductivity were measured with platinum electrodes, and pH was measured with a glass electrode.

During the ALERT project, which extended over several weeks and involved operator-supervised data acquisition, measurements were intermittent due to holidays and weekends. During these periods, the measuring instrumentation remained inactive for extended durations, including overnight and across multiple consecutive days.

**Figure 5:**
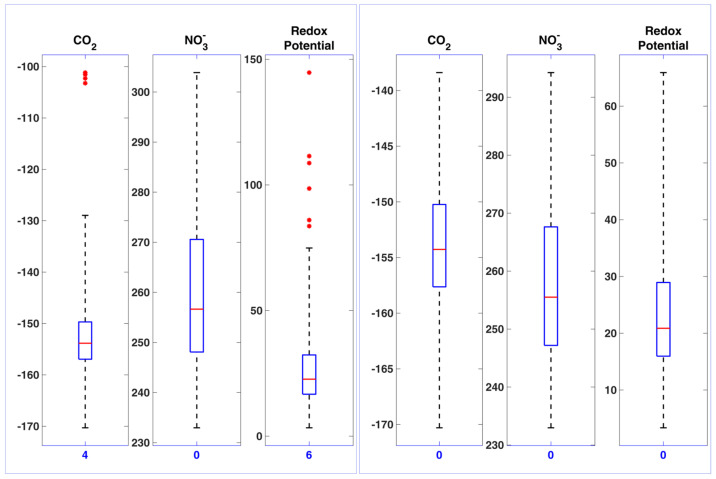
Exemplificative set of measurements to show the impact of daily chemical conditioning on the used sensors. A brief description of the sensors is provided in the text. The milk parameter boxplots on the (**left panel**) are referred to 220 measurements collected during the ALERT Project. Outliers are marked in red, and their number is reported at the bottom of each plot. The (**right panel**) shows the same milk samples with the only exception from the first two samples of each day (188 samples remaining in total), to account for the daily “chemical conditioning”. All reported sensor figures are expressed in mV.

Data analysis revealed an unexpectedly high number of outliers for certain sensors (Figure 5, left panel). Initially, anecdotal evidence suggested that the issue might stem from the choice of electrode conservation solution—a concern reported in the existing literature [59].

However, subsequent investigation showed that pauses during holidays or weekends did not impact the measurement process itself, based on the average of twice-daily measurements (morning and afternoon). The cause for the unexpected sensor behavior was later identified: more than 90% of outliers originated from the first measurements taken each day. It was determined that certain sensors require not only an electronic warm-up period—as specified by the manufacturer and already integrated into the measurement protocol—but also a period of “chemical conditioning” of the sensor membrane/ milk interface when first immersed in milk.

This additional requirement was shown to be met by performing two conditioning cycles, each lasting approximately five minutes, on raw milk samples prior to start of daily measurements.

**Figure 6:**
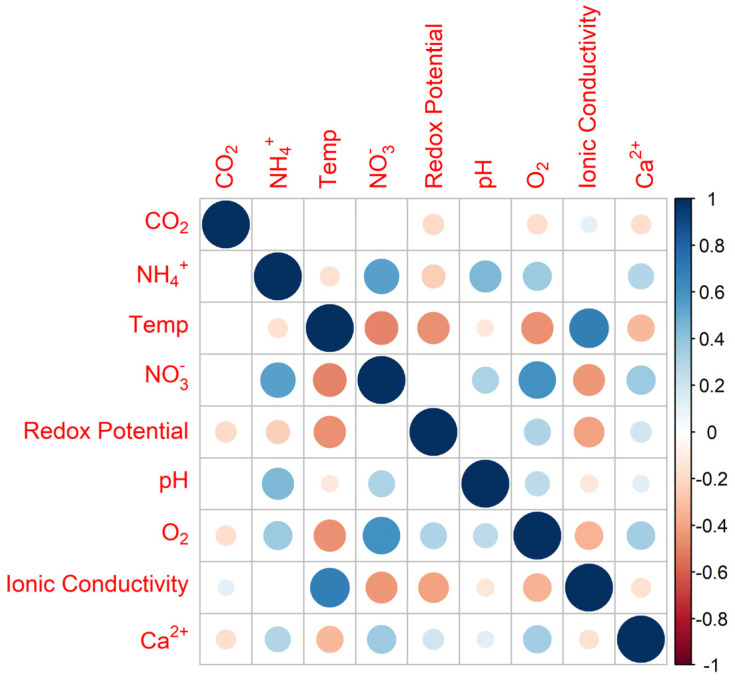
Correlation analyses on all milk parameters measured by BEST platform.

The impact of chemical conditioning is illustrated in the right panel of Figure 5, where the number of outliers in sensor measurements is noticeably reduced compared to the corresponding raw data shown in the left panel. Non-parametric basic statistics revealed significant differences for the median values of each sensor before and after excluding the first daily measurement (Wilcoxon Signed Rank Test, *p* < 0.05), as well as a significant reduction in variability for CO_2_ and redox potential (two-sample test for variance, *p* < 0.05). An analysis of the correlations among the parameter measured by BEST sensors is presented in Figure 6 and Table 3. As shown in Figure 6, most sensor outputs exhibited only weak correlations (larger circles indicate stronger correlations; see scale on the left). In cases where no circle is shown, the correlation did not reach statistical significance (*p* > 0.05). Only a few sensor pairs—such as ionic conductivity/temperature and O_2_/NO_3_^−^—demonstrated moderate correlations (r > 0.6).

### 3.2. The Need for a Predefined Lab Measurement Protocol Including an On-The Field Clear Sampling Plan and Strategy

When validation occurs in different labs—such as those involved in separate aspects of milk process analytics—a common, detailed measurement protocol is essential. This protocol should include instructions for sample handling, hardware setups, selection and use of biochemical reagents, and any required manual or automated operating procedures. The need for such standardization is especially critical when validation procedures must be replicated across multiple laboratories.

Operating procedures should provide a detailed description of all steps required to perform the measurements, including the timing of each step and reference intervals for both intermediate and final output results.

The measurement process should follow a detailed procedure covering all activities from sample collection to analysis. This procedure should include instructions for sample preparation, monitoring, and recording sample and environmental temperature, and should timestamp all relevant steps. When a sample needs to be analyzed using different measurement platforms, a data recording procedure must be in place ensure measurements from the same sample can be easily matched. If necessary, a timestamp synchronization procedure should also be implemented. Standardizing the measurement protocol could further facilitate the integration of sensor-based technologies with confirmatory analyses like MS, to monitor milk components and contaminants. This could be achieved either by pre-screening samples or by enhancing the interpretation of MS data.

Additionally, since on-field data collection is highly resource-intensive, a detailed sampling plan should be developed and agreed upon by all parties involved to optimize the available resources. This is especially important when dairy farmers or untrained operators are involved in the data collection process. In such cases, formalized training and clear procedures should be implemented.

To ensure consistent analysis of milk samples over time, the measurement process must be standardized, traceable, repeatable, and reliable, yielding high-quality data for analysis. A suitable database should be designed to organize the recorded results and various elements of the measurement protocol effectively. All relevant information should be recorded and timestamped. Typical elements of a measurement protocol are detailed in Table 4.

A dedicated recording of all these elements will enhanche the reliability and reproducibility of subsequent analyses over time.

Databases should be constructed using a technique that is both functional and familiar to all members of the data analysis team. Ideally, relational databases should be implemented, as they store and provide access to related data. In a relational database, data is organized into tables (also known as relations), consisting of rows and columns. Each table has a unique key that identifies the data in each row, and tables can be linked to each other using foreign keys. Relational databases are widely used due to their robustness, scalability, and ability to handle complex queries, offering efficient data retrieval, insertion, updating, and deletion—thus providing flexibility during data analysis.

Another general suggestion is to avoid using spreadsheets for data sharing or analysis, as they may lead to inadvertent data modifications. Instead, it is preferable to use formats that can be easily exported and imported into the chosen data analysis environment, such as comma-separated values (CSV) files.

Managing the physiological variability in milk parameters is crucial in the context of data collection and preliminary analysis. Milk sample analytes can exhibit significant variability, including inter- and intra-individual differences, as well as seasonal and temporal fluctuations. For individual cow milk samples, it is recommended to routinely record the animal health’s status and any treatment administered in the database. To address this challenge effectively, it is essential to perform a substantial number of repeated measurements across a relevant sample size. Early repeated measurements can help identify unexpected variability or measurement instabilities, guiding necessary adjustments to the measurement plan. Limiting data collection to a period of 1–2 months can help mitigate the effect of seasonal variability.

The ability of the measurement setup to detect process deviations should be demonstrated in advance. This can be achieved by artificially introducing controlled deviations at each step of the measurement chain and observing their final effects. With appropriate precautions, this validation can take various approaches—for example, by including animals with known health issues in the sampling process or conducting spike testing on milk samples. Throughout the dairy supply chain, milk samples are collected at various points to check for antibiotics, contaminants such as dyes, preservatives, additives, pesticides, or even added water.

To elucidate underlying causative factors, it is essential to identify and interpret any linear trends in milk sample measurements. Such trends should only be removed after a thorough understanding of the biochemical, chemo-physical, or physiological processes that drive them. Additionally, because milk parameters often exhibit a non-normal distribution, it is critical to assess the normality of each parameter prior to statistical analysis. When normality assumptions are not met, non-parametric statistical methods should be employed. Non-normally distributed parameters may produce outliers, making outlier detection a vital step in data preprocessing, as outliers can significantly affect the performance and reliability of statistical models. It is recommended to use multiple outlier detection methods—statistical techniques designed to identify data points that deviate substantially from the majority of the dataset. Failure to address outliers can distort the outcomes of statistical analyses or data mining efforts.

Outlier detection approaches can be univariate or multivariate. Univariate methods analyze individual variables independently, flagging observations that deviate significantly within a single dimension. In contrast, multivariate methods consider multiple variables simultaneously, detecting observations that diverge from the overall multivariate pattern. These methods are particularly suited for complex datasets where relationships between variables must be accounted for.

Moreover, artificial outliers can be introduced into the dataset using results from spike testing on milk samples. This allows for an evaluation of the effectiveness and robustness of the selected outlier detection and removal procedures.

### 3.3. Dealing with the Unexpected: Getting the Most from Instrument Prototypes

In previous sections, several recommendations were provided to address measurement challenges associated with introducing new instrumentation into technologically complex environments, such as a dairy farm. However, based on our experience, an additional and significant factor impacted our ability to meet project timelines: unexpected hardware failures. These failures, occurring at multiple points during the project, led to substantial delays.

When working with internally developed prototypes, equipping teams with multiple identical units can offer substantial benefits. This strategy not only expedites operations in the event of hardware failure but also minimizes the need for urgent repairs, which can divert critical resources from development activities. Furthermore, maintaining multiple prototypes enables the replication of field issues in controlled laboratory conditions, often facilitating faster diagnosis and resolution of problems.

To further mitigate operational risks, spare materials and replacement parts should be readily accessible. This includes components that are prone to failure as well as those whose replacement would be delayed due to administrative or logistical constraints. While these measures may require additional financial investment, they can be offset by tangible savings through reduced downtime, streamlined logistics, and improved workflow continuity.

In multi-site field trials, the use of multiple prototypes also aids in distinguishing site-specific anomalies—such as those related to operator behavior—from actual technical faults. This distinction is crucial for accurate troubleshooting and for refining both the hardware and the implementation process.

## 4. Discussion

Increased consumers awareness of food consumption and the growing demand for healthier and sustainable food options are key drivers for the broader adoption of food quality and safety controls, beginning at the stage of primary production. However, as with other areas of primary food production, the dairy farm environment is not suitable for accommodating high-end laboratory techniques that are considered the gold standard for detection—such as MS and MS/MS. The use of these sensitive and selective analytical methods is associated with high costs, time-consuming procedures, and the need for complex laboratory infrastructure. Moreover, they require trained personnel with specialized technical skills to conduct the analysis.

To address the current demand for rapid, cost effective, user-friendly, and portable diagnostic solutions, there is a growing interest in on-farm, (semi)automated POC tests capable of operating under harsh farm conditions. Sensor-based technologies present new opportunities for real-time analysis, contributing to improved herd management, enhanced animal welfare, and increased product safety. These advances collectively support greater efficiency, sustainability, and traceability within the dairy farming sector [9].

Electrochemical, optical, and microfluidic devices are being developed as practical solutions to meet the growing demand for rapid diagnostics, particularly in agriculture and the dairy industry, where changes in milk composition are increasingly recognized as early indicators of safety and product quality [60,61]. These emerging devices must contend with the complexity of the milk matrix, which is rich in fat globules and proteins that can interact with the surfaces of fluidic components and sensors. Over time, such interactions can lead to the deposition of milk constituents, the formation of fouling layers, and a consequent decline in sensor performance. For these reasons, translating measurement and detection technologies from the laboratory presents several practical challenges. In particular, meeting the requirements for sample preparation and ensuring procedural robustness—especially in terms of repeatability under harsh environmental conditions—is considerably more difficult outside a controlled laboratory setting. The collection, preparation, and analysis of raw cow’s milk samples on an operating dairy farm, using new or adapted technologies, can prove to be significantly more complex than initially anticipated.

The experiences discusses in this work, based on two field measurement campaigns conducted by the authors as part of the ALERT and the MOLOKO projects [38,40,41,54] revealed several weaknesses (internal factors) and threats (external factors related to the specific environment) inherent in the overall approach. The most critical issues have been outlined in the Results section, along with practical recommendation. Some of these suggestions are applicable only to similar experimental setups, while others possess broader relevance.

The following bullet points summarize the key findings and recommendations, emphasizing what is needed:

- A comprehensive understanding of sensors and instrumentation behavior (see Section 3.1): Specifications and guidelines provided by design and testing laboratories are essential; however, they are not sufficient on their own. Each sensor must be fully characterized under actual working conditions once installed in the field. This characterization should include, among other factors, calibration procedures, warm-up time, and chemical conditioning requirements.
- The establishment of a predefined, detailed laboratory measurement protocol: Such a protocol would benefit both in-field measurements using sensor-based technologies and their integration with laboratory-based analytical techniques, such as MS. Sensor-based devices can complement gold-standard methods by serving as rapid, preliminary screening tools, thereby reducing the number of samples requiring labor-intensive and time-consuming MS analysis. This approach not only enhances the overall efficiency and accuracy of contaminant detection but also optimizes workflow, improves resource allocation, and accelerates decision-making in food safety assessments.
- The inclusion of a clear sampling plan and field measurement strategy in the laboratory protocol (see Section 3.2): The protocol should be iteratively tested and validated in advance, and must be shared with, and agreed upon by, all stakeholders involved in data management and analysis. At a minimum, it should address key factors such as environmental conditions, ensuring that the sampling process is robust, reproducible, and suitable for integration with both field and laboratory procedures.
- The development of a comprehensive sampling plan and associated operational framework: The sampling plan should clearly define the overall strategy, including sample size, selection of sampling locations, and sampling frequency. It must specify procedures for sample collection, handling, and storage to ensure consistency and integrity. In addition, the measurement methods to be used should be explicitly described. The protocol should also identify the personnel involved, detailing their roles and required training. Furthermore, data recording and management must be addressed through the design, construction, and testing of a structured database, accompanied by a user manual to ensure proper and consistent use.
- A quality control preparedness to address unexpected instrument behavior and failures: In field campaigns—particularly when using internally developed prototypes or deploying new instrumentation in technologically challenging environments such as dairy farms—unexpected hardware failures can occur. To ensure the continuity and success of measurements, it is essential to maintain duplicates of entire measurement systems and/or critical hardware components. This redundancy supports rapid recovery from failures and minimizes disruptions during data collection.

## 5. Conclusions

In this paper, we present a collection of practical observations and recommendations derived from two research projects—ALERT and MOLOKO—which utilized sensor-based technologies, specifically electrochemical, optical, and microfluidic devices, for real-time analysis of in-farm milk sampling. These insights cover a range of topics, including sensor behavior in milk matrixes, milk sampling strategies, data processing challenges (such as calibration drift and signal noise), procedural and protocol-related issues, and logistical considerations relevant to project execution.

In both projects, significant limitations stemmed from an initial underestimation of the human and technical resources required to manage the complex and unpredictable conditions of dairy farm environments. These challenges include technical failures, unexpected instrument behavior, unstable data communication, and the high variability of raw milk properties and sampling conditions. As a result, the scope of sampling could not be expanded as planned —either in terms of sample quantity or duration.

Notably, data collection from animals in critical health conditions was not possible. Although such data could have enriched the research datasets, compliance with food safety regulations prevented the inclusion of milk from unhealthy animals, due to its exclusion from the official milk production and supply chain. Despite these challenges, we hope the observations reported here will assist others in accelerating data collection, analysis, and validation efforts under similarly demanding field conditions. Building on the outcomes of the ALERT and MOLOKO projects, several future directions emerge. For both the BEST and MOLOKO systems, expanding the range of detectable parameters and advancing sensor technologies—such as through the development of novel sensing materials—would be highly beneficial. Furthermore, integrating a machine learning approach into the BEST platform could significantly enhance outlier detection, improve analytical accuracy and speed, and potentially enable predictive modeling to anticipate quality and safety issues in milk production.

## Figures and Tables

**Table 1:** Analytical characteristics of sensors integrated into the BEST platform.

Sensor	Measurement Range *	Other Specifications *
Ca^2+^ ISE	0.02–4010.00 mg/L	Sensitivity: +25 to +30 mV per decade change in ion activity
NH_4_^+^ ISE	0.9–9000.0 mg/L	Sensitivity: +54 to +60 mV per decade change in ion activity
NO_3_^−^ ISE	0.4–62,000.0 mg/L	Sensitivity: +52 to +58 mV per decade change in ion activity
Cl^−^ ISE	1–35,500 mg/L	Sensitivity: +54 to +60 mV per decade change in ion activity
CO_2_ gas-permeable membrane electrode	8.80–880.20 mg/L	Accuracy: ±1.95 mg/L
O_2_ luminescent gas-sensitive probe	8 µg/L to saturation	Accuracy: ±(1% of the reading + 8 µg/L)
pH sensor	0 to 14 pH units	Accuracy (pH units): ±0.1 pH units.
Redox sensor	−2000 to +2000 mV	Accuracy (mV): ±10
Ionic conductivity sensor	0 to 20 mS/cm	Accuracy ± 0.5% of the reading
		Accuracy (°C): ±0.10 °C
Temperature sensor	−100 to +500 °C	

* Data provided by the sensor vendor/manufacturer.

**Table 2:** Comparison, similarities, and advantages of the ALERT and MOLOKO projects.

Feature	Alert Project	MOLOKO Project
Primary focus and scope	Monitoring the quality, health, and traceability of bovine milk	Development of a miniaturized optical biosensor for fast and multiplex plasmonic detection of milk quality and safety parameters.
Technology focus and analytical approach	Using an array of targeted selective detection surfaces and integrated optical and electrochemical sensors (the patented BEST system) for the continuous monitoring of various chemical and physico-chemical parameters in milk, including inorganic ions and gases.	Miniaturized optical biosensor focus on specific analytes using plasmonic sensing by integrating organic optoelectronic devices and nanostructured plasmonic grating; use of a multiplex immunoassay and recombinant antibodies
Parameters measured	Free inorganic ion fraction (Ca^2+^, NH_4_^+^, NO_3_^−^, Cl^−^), dissolved gases (O_2_, CO_2_), ionic conductivity, pH, redox potential, temperature.	Lactoferrin, streptomycin; β-Casein A2 vs. β-Casein A1 (discriminated using a multiplex immunoassay); *Staphylococcus aureus* Enterotoxin A (using recombinant antibodies—patent pending)
Innovation	Continuous monitoring from farm level for early anomaly identification. Definition of integrated markers for specific condition assessment. HACCP-like multi-sensor early warning system for identification and management of anomalies.	Integration of plasmonic sensing with organic optoelectronics; miniaturized optical biosensor for real-setting analysis.
Application and outcomes		
	Self-control activity of the milk supply chain; support for precision livestock farming; building control charts; risk management and traceability.	Potential for point-of-care (POC) systems; accurate and low-cost analytical detection; integration of MOLOKO sensor into milking machines & milking process.

**Table 4:** Typical elements of a measurement protocol.

Elements of a Measurement Protocol	Meaning and Examples
Environmental conditions	Conditions under which measurements should be taken considering the use case and including any environmental control or recording of environmental variables necessary during the measurements
Sampling plan	Overall strategy, including sample size, locations and frequency, how samples should be collected, handled, and stored
Measurement methods	Techniques and instruments to be used for measurements, including calibration and conditioning procedures
Personnel and training	Identification of the laboratory (for offline analyses), personnel/operator responsible for carrying out the measurements, the training and qualifications required for personnel
Data record and management	Guidelines for recording data, including formats and units of measurement, the procedures for data storage, organization, management, and traceability
Quality control	Measures to be implemented to ensure data integrity, including procedures for routine checks and calibration of instruments.

**Table 3:** Statistical analysis table for milk samples and milk parameters measured by BEST platform. The first two samples of each day are excluded (Figure 5, right panel) to account for the daily “chemical conditioning”. SD—standard deviation. IQR—interquartile range. CV—coefficient of variation. Appropriate measures of dispersion are CV for when sensors’ data pass normality tests (Shapiro, last column) or IQR/median otherwise.

Parameter (Unit)	N	Mean	SD	CV	Median	IQR	IQR/Median	Normality
CO_2_ (mV)	188	−153.78	6.11	−0.04	−154.27	7.4	0.05	yes
NH_4_^+^ (mV)	188	253.43	2.92	0.01	253.4	4.64	0.02	yes
Temperature (°C)	188	29.42	1.91	0.07	29.08	3.09	0.11	no
NO_3_^−^ (mV)	188	258.11	14.23	0.06	255.51	20.45	0.08	no
Redox potential (mV)	188	23.62	10.98	0.46	20.88	12.98	0.62	no
pH (pH units)	188	6.56	0.08	0.01	6.56	0.11	0.02	yes
O_2_	188	5.69	0.25	0.04	5.73	0.33	0.06	no
Ionic conductivity (mS/cm)	188	6.44	0.49	0.08	6.42	0.61	0.09	yes
Ca^2+^ (mV)	188	409.46	3.81	0.01	409.31	5.82	0.01	yes
Cl^−^ (mV)	188	103.11	6.68	0.06	102.28	8.96	0.09	yes

## Data Availability

The raw data supporting the conclusions of this article will be made available by the authors on request.

## Abbreviations

The following abbreviations are used in this manuscript:

MDPI	Multidisciplinary Digital Publishing Institute
MS	Mass Spectrometry
MS/MS	tandem Mass Spectrometry
ELISA	Enzyme-Linked Immunosorbent Assay
GC	Gas Chromatography
HPLC	High-Performance Liquid Chromatography
qPCR	quantitative Polymerase Chain Reaction
CCP	Critical Control Point
POC	Point of Care
POPA	Point of Particular Attention
SEA	*Staphylococcus aureus* enterotoxin A
ISE	Ion Selective Electrode
CSV	Comma Separated Values
